# Spontaneous Aortic Rupture: A Case Report

**DOI:** 10.5811/cpcem.1349

**Published:** 2023-11-08

**Authors:** Eshaan J. Daas, Coleman S. Cowart, Amanda Balmages, Ryan Roten

**Affiliations:** Desert Regional Medical Center, Department of Emergency Medicine, Palm Springs, California

**Keywords:** *acute aortic syndrome*, *aortic rupture*, *throat pain*, *case report*

## Abstract

**Introduction:**

Acute aortic syndrome (AAS) includes the disease processes of aortic dissection, penetrating atherosclerotic ulcer, and intramural hematoma. This case demonstrates an atypical presentation of the disease and offers approaches to potentially prevent missed diagnoses.

**Case Report:**

An 87-year-old female with hypertension and Alzheimer’s dementia presented to the emergency department with stable vital signs and a chief complaint of throat pain. Initial work-up was significant for ischemia on electrocardiogram and elevated troponin. Computed tomography of the soft tissue neck revealed evidence of a ruptured aorta.

**Conclusion:**

Aortic rupture is a fatal complication of AAS. In an elderly patient with a history of hypertension, ischemic changes on electrocardiogram, and nonspecific pain, AAS should be on the emergency physician’s differential even in the setting of a benign or limited history and exam.

CPC-EM Capsule What do we already know about this clinical entity?

*Acute aortic syndrome involves multiple pathologies including penetrating atherosclerotic ulcer, which accounts for 42% of fatal cases of aortic rupture.*

What is the major learning point?

*Diagnosing aortic injury in patients presenting with non-specific symptoms is challenging and requires a blend of clinical gestalt and targeted diagnostic work-up.*

How might this improve emergency medicine practice?

*Symptomatology may be benign even in life-threatening situations of aortic injury, thereby increasing the importance of maintaining an inclusive differential.*

## INTRODUCTION

**Figure 1.**
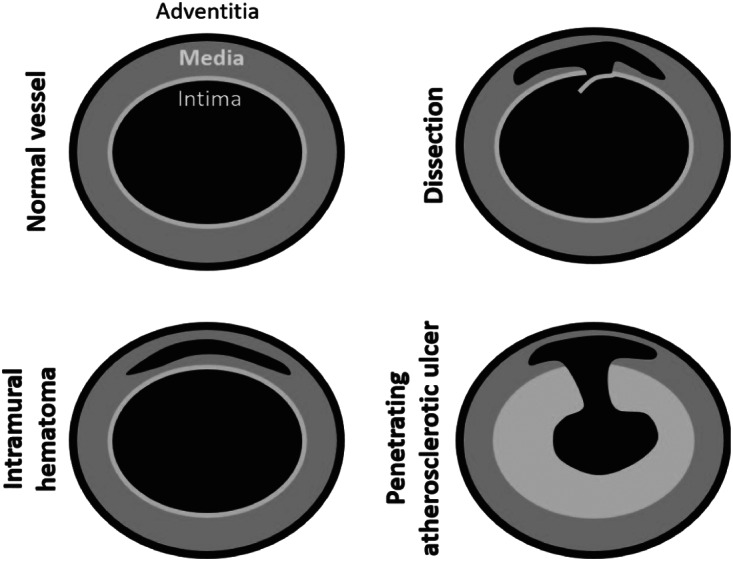
Acute aortic syndrome pathophysiology with illustration of the layers of the aorta.^6^

Acute aortic syndrome (AAS) is a medical diagnosis that includes multiple thoracic and abdominal aortic pathologies.^1^ Penetrating atherosclerotic ulcer (PAU) results from an atherosclerotic aortic plaque invading through the internal elastic lamina into the aortic media (Figure 1). Intramural hematoma (IMH) results from the rupture of vasa vasorum, small vessels that provide oxygen to the arterial wall, which subsequently causes bleeding into the outer layers of the aortic media.^2^ Aortic dissection (AD) includes the pathophysiology identified in IMH with additional disruption of the intima.^3^ Current literature suggests PAU and IMH account for 2.3–7.6% and 6–10% of cases of AAS, respectively.^2^ Aortic rupture is a fatal complication of all three entities; however, the risk is greatest with PAU with a rate of 42%, followed by IMH at a rate of 35%.^4^ Current estimates indicate an incidence of AD between 2.6–3.5 cases per 100,000 person-years with the majority of patients being males in their sixth decade of life according to the International Registry of Acute Aortic Dissection.^5^ The sudden onset of severe, sharp pain was the single most common presenting complaint.

In this case, we present a female patient in her ninth decade of life with dementia at baseline, endorsing a chief complaint of throat pain. Given the wide variety of clinical presentations and high mortality rate associated with aortic rupture, diagnostic strategies include contrast-enhanced spiral computed tomography (CT), transesophageal echocardiogram, and magnetic resonance imaging when clinical suspicion is high. However, in cases with history limited by memory impairment and non-specific symptomatology unexplained by physical examination, an algorithmic approach to diagnosis and treatment becomes difficult. The case report presented here details the late identification of a life-threatening aortic rupture in an emergent setting with weak initial evidence to suggest the diagnosis.

## CASE REPORT

An 87-year-old female with a past medical history significant for hypertension and Alzheimer’s dementia presented to the emergency department (ED) with her daughter, endorsing throat pain that began upon awakening in the morning. The patient’s daughter reported that her mother had consumed a regular meal without difficulty the night prior. The patient also reportedly had consumed caustic substances in the past including cleaning supplies, confusing them for water/juice. According to the patient and her family, she had not had any upper respiratory infection symptoms such as cough and fever. The patient also specifically denied chest pain, shortness of breath, abdominal pain, and vomiting. She was hemodynamically stable and afebrile on presentation including blood pressure of 138/87 millimeters of mercury and heart rate of 82 beats per minute with an oxygen saturation of 98% on room air. On physical examination, airway was patent with no erythema or swelling of the oropharynx, no pulse deficits in the extremities, lymphadenopathy, or neurological deficits with the exception of baseline orientation to self only.

**Image 1.**
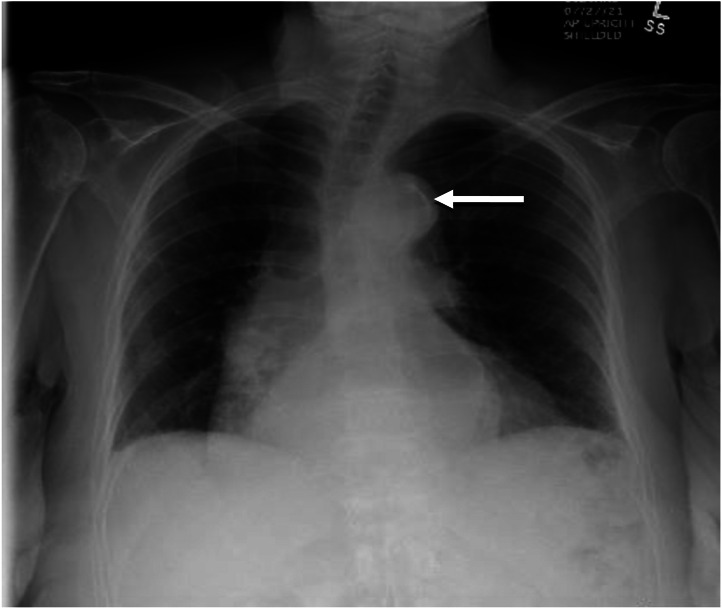
Chest radiograph showing mild left costophrenic blunting and atherosclerotic calcification of the aorta (arrow).

Laboratory work-up was significant for elevation in serial troponin from 0.048 to 0.086 nanograms/milliliter (ng/mL) (reference range: 0.000–0.034 ng/mL). Initial electrocardiogram (ECG) showed sinus bradycardia at a rate of 43 beats per minute with T-wave inversions in leads I and aVL, more pronounced compared to prior studies. Chest radiograph revealed atherosclerotic calcifications of the aorta and mild blunting of the right costophrenic angle without widening of the mediastinum. There was less than 5 millimeters of separation of the intimal calcification from the outer aortic border, which was similar to findings in a previous radiograph (Image 1).

Given the patient’s ECG changes and elevated troponin, admission for acute coronary syndrome work-up was pursued. Additionally, the history of previous caustic ingestions in the setting of severe dementia and presenting complaint of throat pain raised clinical suspicion for corrosive upper gastrointestinal tract injury. A CT soft tissue neck with contrast was obtained to evaluate for edema, stranding, stricture formation, and associated fistulous complications.

**Image 2.**
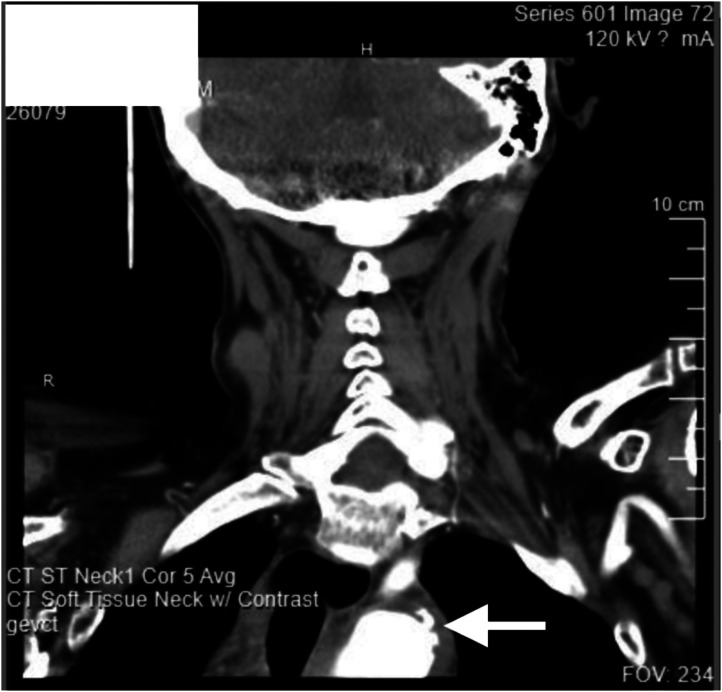
Computed tomography soft tissue neck with arrow indicating the contrast extending into the posterior wall of the proximal descending aorta consistent with a penetrating atherosclerotic ulcer in coronal view.

During evaluation, the patient became severely bradycardic, apneic, and eventually unresponsive. Resuscitation efforts were initiated and discontinued shortly thereafter, following clarification from the patient’s daughter of “Do Not Resuscitate/Do Not Intubate” code status. At the conclusion of the code, radiology called to inform of fluid in the superior mediastinum suggestive of aortic leak and contrast extending into the posterior wall of the proximal descending thoracic aorta suggestive of penetrating atherosclerotic ulcer (Image 2).

## DISCUSSION

As documented in emergency medicine literature, most case reports discuss aortic injuries secondary to traumatic mechanisms. In these instances, routinely obtained contrast-enhanced imaging of the chest and abdomen lead to the prompt identification of pathology even in the absence of hemodynamic instability. The limited case reports of spontaneous aortic rupture recommend that physicians maintain a high clinical suspicion for aortic injury in a bimodal age distribution to include younger patients with connective tissue disease and older males with chronic hypertension and other atherosclerotic risk factors. Greater than 50% of patients describe tearing/ripping pain in the chest radiating to the back with non-specific physical exam findings, which may include pulse discrepancies, neurologic deficits, or new-onset murmurs. The imaging modality of choice for AAS is CT angiogram (CTA).^7^ Treatment varies depending on the patient’s symptoms, location of injury, and whether the patient has a singular disease process vs multiple manifestations of AAS with options ranging from medical management focused on blood pressure control to surgical intervention with open and endovascular approaches available.

In the case described above, the patient presented hemodynamically stable with an atypical chief complaint of throat pain with a primary risk factor of hypertension. Given her advanced dementia, she was unable to provide reliable chronological and qualitative information regarding her symptoms. Work-up was remarkable for ischemic changes on ECG and elevation in troponin. Physicians reached the ultimate diagnosis of aortic rupture incidentally through imaging initially obtained to further investigate potential caustic ingestion as the etiology of the patient’s throat pain. However, at the time of diagnosis, the patient had already become unresponsive and pulseless.

Spontaneous aortic rupture is a diagnosis with high mortality that has great potential to be seen and missed by emergency physicians. Elements of initial work-up in patients with undifferentiated chest/back pain and history of hypertension that can aid in the prompt diagnosis of aortic rupture include calcium sign on chest radiograph (white line of calcium within aortic knob and measure to outer edge of the aortic knob), ischemia on ECG, and widened aortic outflow tract on point-of-care ultrasound.^8^ Abnormalities in the aforementioned diagnostic studies may bolster clinical suspicion for aortic injury and serve as indication for further investigation including CTA of the chest and abdomen.

Admittedly, diagnosis of spontaneous aortic rupture without convincing history and physical elements is difficult to reach. Few cases of aortic dissection presenting as throat pain have been reported in previous literature. In a case from 2004, a 53-year-old male presented to the ED with sensation of retained foreign body in his throat in the setting of known history of cigarette smoking. The patient was discharged following a benign oropharyngeal exam including plain films of the neck and laryngoscopy. Ten hours later, the patient returned and was found to be hypotensive with tachycardia. Imaging of the chest and abdomen was significant for ascending aortic dissection with cardiac tamponade. The patient passed away during an emergent attempt at operative repair.^9^

Similarly, in 2010 a 58-year-old male with a history of hypertension presented with intermittent dysphagia with stable vital signs. Laboratory work-up was largely unremarkable and imaging was not obtained, but the patient was referred to an emergency otolaryngology unit for suspected peritonsillar abscess. Shortly thereafter, the patient expired after being found to have a massive ascending aortic dissection on imaging and cardiac tamponade physiology.^10^ The case presented here and the reports found through literature review share very important elements including initial presentation characterized by throat pain in a patient with atherosclerotic risk factors and normal oropharyngeal exam. Significant consideration in this patient population should be given to a possible diagnosis of AAS. Ultimately, successful management will depend on a clinically broad diagnostic approach and flexible index of suspicion with consideration of risk factors and overall epidemiology of aortic rupture.

## CONCLUSION

Acute aortic syndrome is an umbrella term to describe pathologies involving aortic injury generally seen in patients with a history of trauma or risk factors for atherosclerosis. While a standard for medical and surgical management of the condition has been agreed upon in literature, reaching the diagnosis in select patients can be difficult, as discussed in the case above. Due to the high risk of mortality in patients presenting with aortic rupture, it is important for physicians to consider it in their differential diagnosis based on presentation, history, ECG, and radiologic findings including radiograph and ultrasound.
